# Sex differences in insulin resistance, but not peripheral neuropathy, in a diet-induced prediabetes mouse model

**DOI:** 10.1242/dmm.048909

**Published:** 2021-04-15

**Authors:** Sarah E. Elzinga, Masha G. Savelieff, Phillipe D. O'Brien, Faye E. Mendelson, John M. Hayes, Eva L. Feldman

**Affiliations:** 1 Department of Neurology, University of Michigan, Ann Arbor, MI 48109, USA; 2 NeuroNetwork for Emerging Therapies, University of Michigan, Ann Arbor, MI 48109, USA

**Keywords:** Diabetes, High-fat diet, Insulin resistance, Obesity, Peripheral neuropathy, Sex dimorphism

## Abstract

Peripheral neuropathy (PN) is a common complication of prediabetes and diabetes and is an increasing problem worldwide. Existing PN treatments rely solely on glycemic control, which is effective in type 1 but not type 2 diabetes. Sex differences in response to anti-diabetic drugs further complicate the identification of effective PN therapies. Preclinical research has been primarily carried out in males, highlighting the need for increased sex consideration in PN models. We previously reported PN sex dimorphism in obese leptin-deficient *ob*/*ob* mice. This genetic model is inherently limited, however, owing to leptin's role in metabolism. Therefore, the current study goal was to examine PN and insulin resistance in male and female C57BL6/J mice fed a high-fat diet (HFD), an established murine model of human prediabetes lacking genetic mutations. HFD mice of both sexes underwent longitudinal phenotyping and exhibited expected metabolic and PN dysfunction compared to standard diet (SD)-fed animals. Hindpaw thermal latencies to heat were shorter in HFD females versus HFD males, as well as SD females versus males. Compared to HFD males, female HFD mice exhibited delayed insulin resistance, yet still developed the same trajectory of nerve conduction deficits and intraepidermal nerve fiber density loss. Subtle differences in adipokine levels were also noted by sex and obesity status. Collectively, our results indicate that although females retain early insulin sensitivity upon HFD challenge, this does not protect them from developing the same degree of PN as their male counterparts.

This article has an associated First Person interview with the first author of the paper.

## Abstract (precis)

**Summary:** We established a prediabetic obese mouse model for studying sex dimorphism in peripheral neuropathy. Females retain early insulin sensitivity upon high-fat diet challenge, but develop the same degree of peripheral neuropathy as males.

## INTRODUCTION

Peripheral neuropathy (PN) is a common complication in individuals with prediabetes and diabetes, which can lead to pain, gait instability and even limb amputations (Feldman et al., 2019). Unfortunately, to date, there are no curative PN treatments, and management of individuals with prediabetes and diabetes rests solely on glycemic control, which is only marginally effective for the more prevalent diabetes type, type 2 diabetes (T2D) (Callaghan et al., 2012). Further complicating the search for PN treatments is the differential effectiveness of anti-diabetic drugs between males and females. For example, treatment responses to sulfonylureas are more pronounced in males, who conversely have a lower responsiveness to thiazolidinediones compared to females (Dennis et al., 2018; Kim et al., 2005). When analyzing factors that contribute to a greater decrease in glycated hemoglobin (GHb) in response to insulin therapy, male sex is a significant contributor (Österbrand et al., 2007). Conversely, exenatide and/or dapagliflozin in combination with metformin stimulates greater weight loss in females than in males (Frías et al., 2018). These differential sex-dependent responses to anti-diabetic drugs (Franconi and Campesi, 2014) and drugs in general (Spoletini et al., 2012) boldly underscore the need for a fundamental understanding of sex dimorphism in disease, including prediabetes, diabetes and PN. These sentiments are echoed by a recent National Institutes of Health mandate, which requires preclinical research to consider sex effects (Clayton and Collins, 2014) to promote the development of tailored clinical management strategies.

Animal models that faithfully mirror disease in humans have been instrumental in advancing our understanding of prediabetes, diabetes and PN pathophysiology (O'Brien et al., 2014; Hinder et al., 2017a; O'Brien et al., 2018), and as a platform for testing potential therapeutics or dietary interventions (O'Brien et al., 2020; Hur et al., 2015; Eid et al., 2020). Recently, animal models are being used for investigating potential sex effects (Sorge and Totsch, 2017; Becker and Koob, 2016), including in obesity, diabetes and PN (Zore et al., 2018; Karastergiou and Fried, 2017; Giatti et al., 2019). We previously identified sex differences in PN using the leptin-deficient *ob*/*ob* T2D mouse model (O'Brien et al., 2016). Compared to lean controls, male and female *ob*/*ob* mice developed pronounced metabolic dysfunction and similar nerve conduction velocity (NCV) slowing, a marker of myelinated fiber injury. However, unmyelinated fiber injury was more robust in *ob*/*ob* males compared to females. Although informative, the *ob*/*ob* model is limited due to complete loss of leptin production, a non-physiological phenomenon, which is not part of the pathophysiology of prediabetes or diabetes (Dunmore and Brown, 2013, D'Souza et al., 2017). This weakness is further compounded by leptin levels that differ naturally in males and females (Christen et al., 2018), and are intimately tied to body fat distribution, insulin, energy metabolism, sex hormones and even the immune system (Ahima and Flier, 2000; Havel, 2004), limiting the use of the *ob*/*ob* model in the context of sex dimorphism studies. Moreover, genetic models suffer from the potentially confounding effects of the mutation.

To overcome these limitations, we re-evaluated sex differences in PN using a high-fat diet (HFD) mouse model (Hinder et al., 2017a; O'Brien et al., 2018, 2020; Yorek et al., 2017; Obrosova et al., 2007). In this model, wild-type C57BL/6 mice are placed on a HFD enriched in saturated fatty acids, leading to diet-induced obesity and prediabetes. HFD obese prediabetic mice robustly and consistently develop elevated fasting blood glucose (FBG), impaired insulin sensitivity, hyperinsulinemia, dyslipidemia, and both small- and large-fiber PN (Hinder et al., 2017a; O'Brien et al., 2018, 2020). Studies have shown that the HFD mouse recapitulates many features of human obesity and prediabetes, which frequently occur simultaneously with other components of the metabolic syndrome, e.g. dyslipidemia (Singleton et al., 2005; Brannick and Dagogo-Jack, 2018), and is a faithful model of diet-induced PN (Hinder et al., 2017a). However, these previous studies were limited because they were conducted with only male mice. Herein, we report differences between males and females in metabolic, neuropathic and circulating adipokine parameters in our HFD-induced obesity and prediabetes mouse model, suggesting that it is critical to consider sex differences in preclinical mouse models.

## RESULTS

### Metabolic phenotyping highlights sex dimorphism in HFD and SD mice

**Fig. 1.**
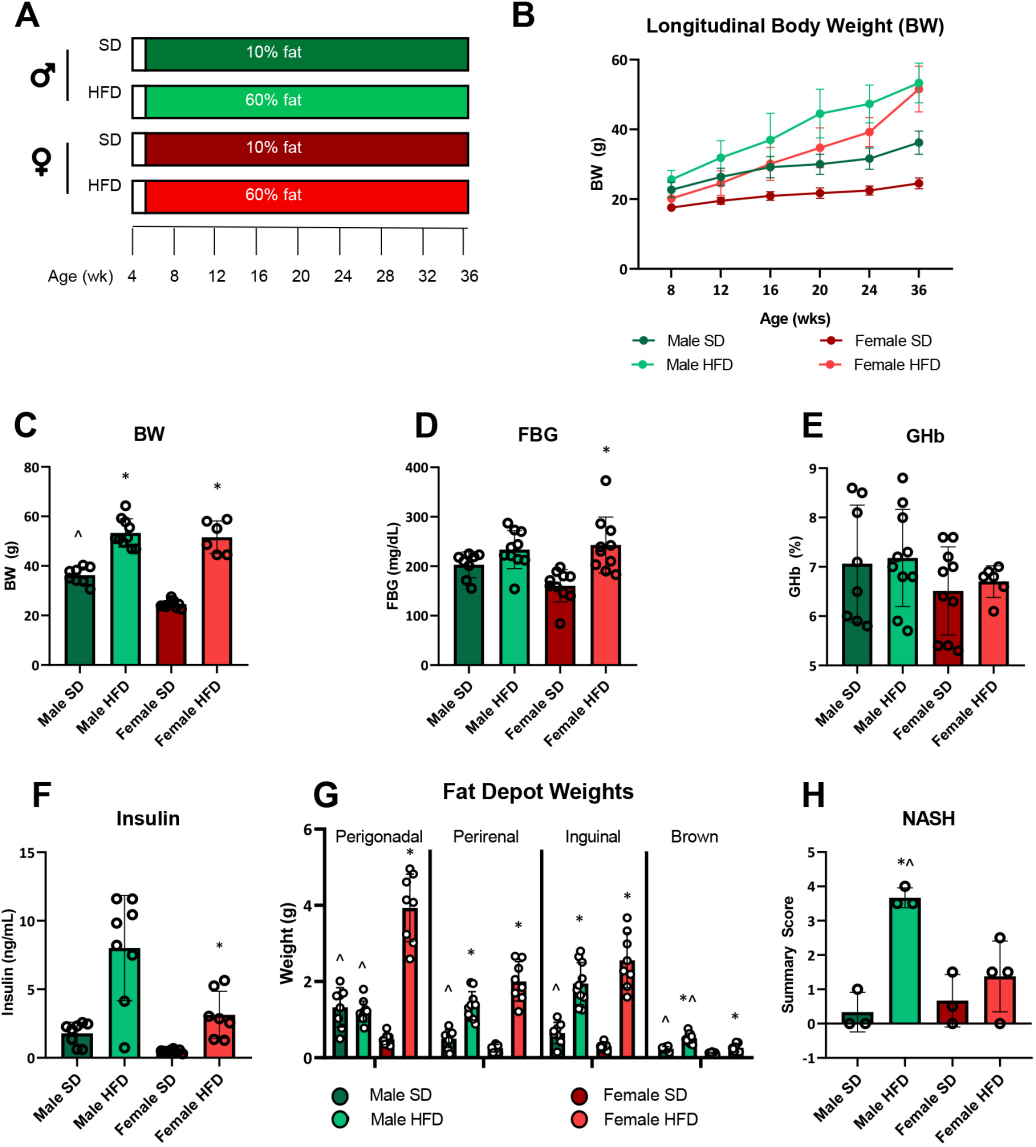
**Terminal (36 weeks) metabolic phenotyping in HFD versus SD male and female C57BL6/J mice.** (A) Study paradigm. (B) Longitudinal body weight (BW), 8, 12, 20 and 24 weeks, *n*=10/group; 16 weeks, *n*=9 SD males, *n*=8 HFD males, *n*=10 SD females, *n*=10 HFD females; 36 weeks, *n*=8 SD males, *n*=10 HFD males, *n*=10 SD females, *n*=6 HFD females. (C) Terminal BW, *n*=8 SD males, *n*=10 HFD males, *n*=10 SD females, *n*=6 HFD females. (D) Terminal fasting blood glucose (FBG), *n*=8 SD males, *n*=10 HFD males, *n*=10 SD females, *n*=10 HFD females. (E) Terminal glycated hemoglobin (GHb), *n*=8 SD males, *n*=10 HFD males, *n*=10 SD females, *n*=6 HFD females. (F) Terminal plasma insulin, *n*=8 SD males, *n*=8 HFD males, *n*=8 SD females, *n*=7 HFD females. (G) Terminal fat depot weights, all fat depots *n*=8 SD males (except brown *n*=7), *n*=10 HFD males, *n*=10 SD females, *n*=8 HFD females. (H) Terminal non-alcoholic steatohepatitis (NASH) score, *n*=3 SD males, *n*=3 HFD males, *n*=3 SD females, *n*=4 HFD females. HFD, high-fat diet; SD, standard diet; individual data points represent individual animals. Statistically significant (*P*<0.05) differences from respective SD controls are represented by * and sex differences within diet by ^. Statistical analyses: data in B and G analyzed by mixed model [restricted maximum likelihood (REML)]; the remaining data analyzed by one-way ANOVA. Data are presented as mean±s.e.m.

Male and female wild-type C57BL6/J mice were fed a HFD or standard diet (SD) starting at 5 weeks of age for 31 weeks until 36 weeks of age (Fig. 1A). During the study course, HFD males gained weight more rapidly, whereas HFD females initially gained weight more slowly, but attained the same weight as HFD males by 36 weeks (Fig. 1B,C). Past 20 weeks, SD males and females consistently weighed less than their respective HFD counterparts, and SD males were always heavier than SD females. At study conclusion, FBG was greater in HFD versus SD females, but was similar in HFD versus SD males (Fig. 1D). Moreover, FBG was slightly elevated in HFD females compared to HFD males, whereas FBG was lower in SD females versus SD males, although neither of these differences was significant. Glycated hemoglobin (GHb) did not differ by sex or diet (Fig. 1E). Terminal plasma insulin at 36 weeks also differed between groups and was elevated in HFD versus SD females, with a trending increase in HFD versus SD males (Fig. 1F). In addition to measuring glycemic control, we also examined differences in fat depots (Fig. 1G), including perigonadal [a visceral white adipose tissue (VAT) depot], perirenal (another VAT), inguinal (flank adipose tissue) and brown adipose tissue from the neck (Tran and Kahn, 2010; de Jong et al., 2015). HFD females had the greatest amount of perigonadal VAT, followed by HFD and SD males, which had similar amounts, and SD females, which had the least. For perirenal VAT, HFD increased depot mass, but this was not affected by sex. Regardless of sex, HFD animals had more inguinal fat; in SD animals, males had larger inguinal depots than females. HFD increased brown adipose tissue depots in both sexes, but to a significantly greater extent in males. SD females had the smallest brown adipose tissue depots. Finally, we assessed fatty liver disease using non-alcoholic steatohepatitis (NASH) summary scores, which were highest in HFD males, followed by HFD females, and lowest in both SD sexes, which did not differ from each other (Fig. 1H).

### Females retain insulin sensitivity early in the course of HFD feeding

**Fig. 2.**
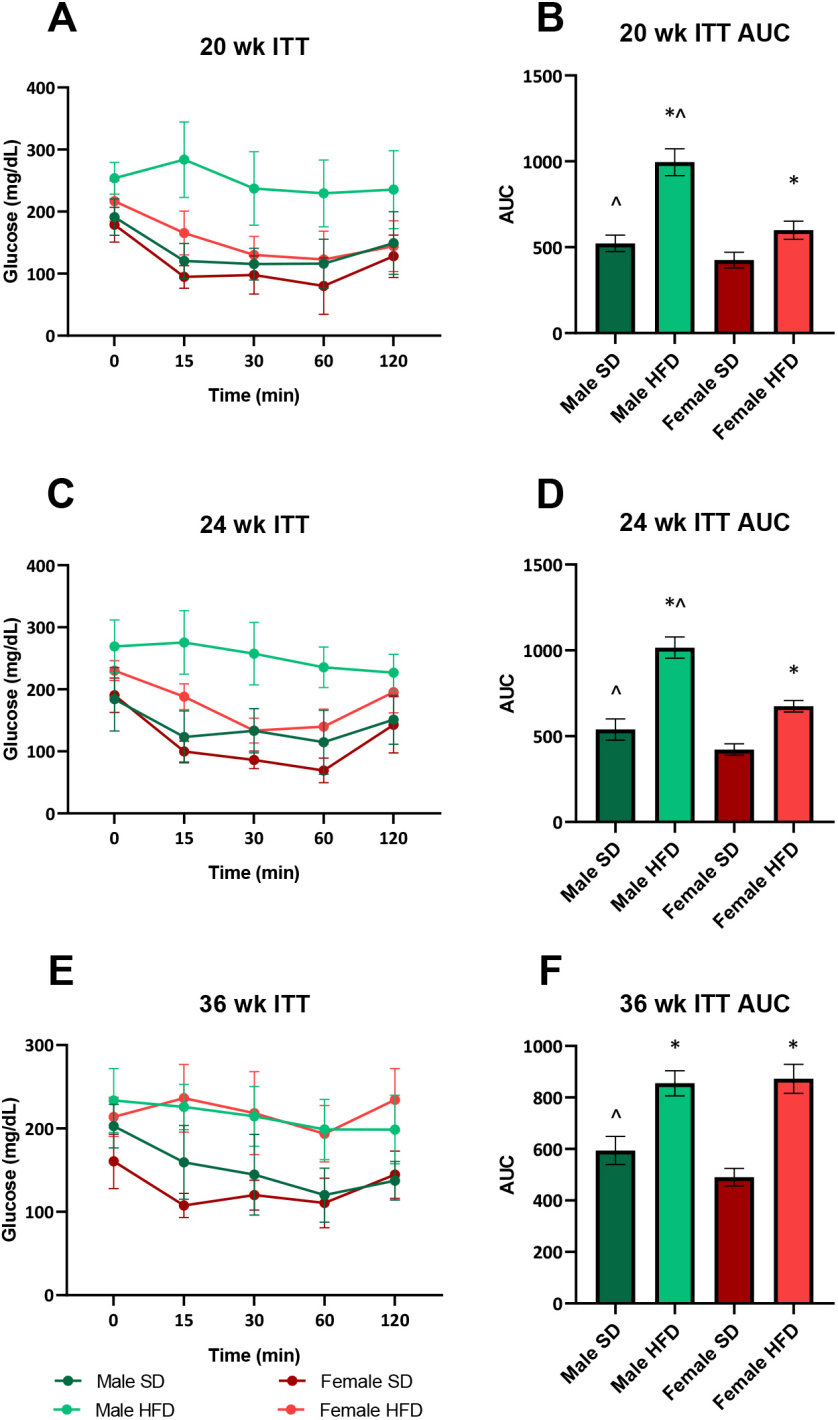
**Longitudinal insulin tolerance tests (ITTs) in HFD versus SD male and female C57BL6/J mice in the 36 weeks study paradigm.** (A) ITT at 20 weeks. (B) ITT area under the curve (AUC) at 20 weeks. (C) ITT at 24 weeks. (D) ITT AUC at 24 weeks. (E) ITT at 36 weeks. (F) ITT AUC at 36 weeks. *n*=10/group at each time point (except SD males at 36 weeks, *n*=8; HFD females at 36 weeks, *n*=7). HFD, high-fat diet; SD, standard diet; individual data points represent individual animals. Statistically significant (*P*<0.05) differences from respective SD controls are represented by * and sex differences within diet by ^. Statistical analyses: data in A, C and E analyzed by repeated measures two-way ANOVA; data in B, D and F analyzed by AUC followed by one-way ANOVA. Data are presented as mean±s.e.m.

During the study course, we monitored insulin sensitivity using insulin tolerance tests (ITTs) (Fig. 2). At 20 weeks of age, i.e. after 15 weeks of HFD feeding, HFD male mice already displayed a blunted response to exogenous insulin (Fig. 2A), whereas HFD females only differed from their respective SD controls at one of the five recorded time points (at 15 min). Although HFD increased the ITT area under the curve (AUC) in both sexes, the extent was greater for males (Fig. 2B). Moreover, the AUC was larger for SD males than for SD females, as well as for HFD males compared to HFD females. At 24 weeks of age, i.e. after 19 weeks of diet, the HFD male ITT responses continued to indicate a greater loss of insulin sensitivity compared to other groups; at this time, HFD females remained insulin sensitive, but not to the degree observed at 20 weeks (Fig. 2C,D). HFD males remained less insulin responsive than their female counterparts, an observation that was paralleled in the SD cohorts. By the 36 weeks study conclusion, i.e. after 31 weeks of diet, HFD females and males had similar loss of insulin sensitivity (Fig. 2E,F), and SD males continued to be less insulin sensitive than their female counterparts. The similar loss of insulin sensitivity, as a marker of insulin resistance (IR), in HFD males and females is corroborated by their fasting plasma insulin levels, which were also similar and higher than their respective SD controls (Fig. 1F).

### Longitudinal neuropathy phenotyping identifies sex differences in thermal sensitivity

**Fig. 3.**
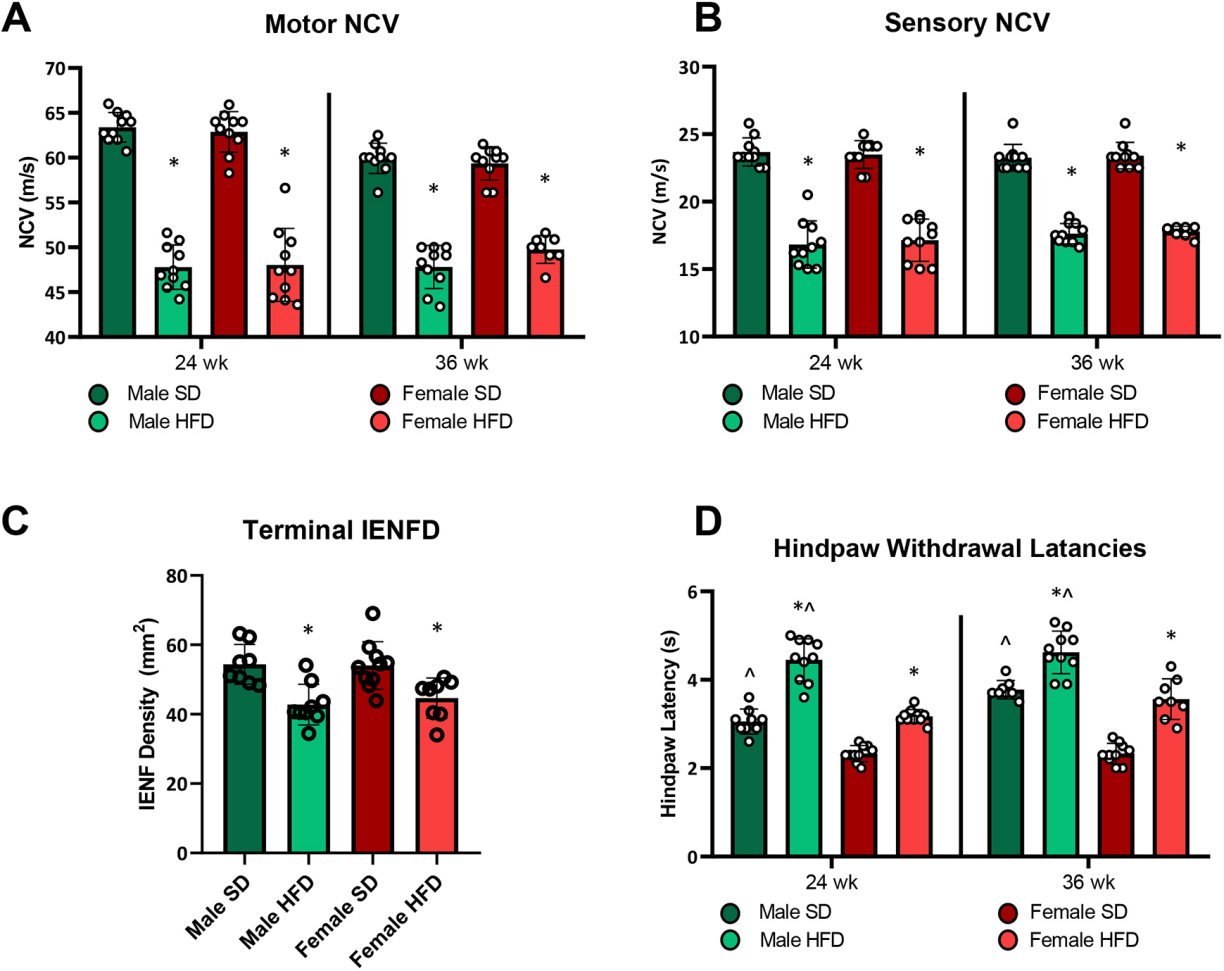
**Longitudinal neuropathy phenotyping in HFD versus SD male and female C57BL6/J mice.** (A) Motor nerve conduction velocities (NCVs), 24 weeks, *n*=10/group; 36 weeks, *n*=10 SD males, *n*=10 HFD males, *n*=10 SD females, *n*=8 HFD females. (B) Sensory NCVs, 24 weeks, *n*=10/group; 36 weeks, *n*=10 SD males, *n*=10 HFD males, *n*=10 SD females, *n*=8 HFD females. (C) Terminal 36 weeks intraepidermal nerve fiber densities (IENFD), *n*=8 SD males, *n*=9 HFD males, *n*=10 SD females, *n*=8 HFD females. (D) Hindpaw withdrawal latency, 24 weeks, *n*=9 SD males, *n*=10 HFD males, *n*=10 SD females, *n*=10 HFD females; 36 weeks, *n*=8 SD males, *n*=10 HFD males, *n*=10 SD females, *n*=8 HFD females. HFD, high-fat diet; SD, standard diet; individual data points represent individual animals. Statistically significant (*P*<0.05) differences from respective SD controls are represented by * and sex differences within diet by ^. Statistical analyses: data in A, B and D analyzed by mixed model (REML); data in C analyzed by one-way ANOVA. Data are presented as mean±s.e.m.

Over the study period, longitudinal PN phenotyping was conducted at 24 and 36 weeks of age (i.e. following 19 and 31 weeks of diet, respectively), which consisted of motor and sensory NCVs and hindpaw thermal latencies. Terminal intraepidermal nerve fiber density (IENFD) was assessed at the 36 weeks study conclusion. NCV evaluates large-fiber function, and is a measure of myelinated nerve impulse propagation, which is slowed in PN. IENFD histologically assesses small, unmyelinated C-fibers in the epidermis, i.e. small-fiber function, which decreases in PN. IENFD loss can involve, among other nociceptors, loss of thermoreceptors with consequent impairment in response to thermal stimulus. We have previously reported that NCVs slow, IENFDs decrease and hindpaw thermal latencies lengthen upon PN progression (O'Brien et al., 2020). Motor and sensory NCVs were slower in HFD versus SD mice, independent of sex (Fig. 3A,B), at both time points. IENFDs at 36 weeks, like NCVs, were lower in HFD versus SD animals and were without sex differences (Fig. 3C). At 24 and 36 weeks, HFD males and females had prolonged thermal latencies compared to SD animals, and HFD male thermal latencies were more delayed than those of HFD females (Fig. 3D).

### Delayed metabolic dysfunction in HFD females does not affect early PN measures

**Fig. 4.**
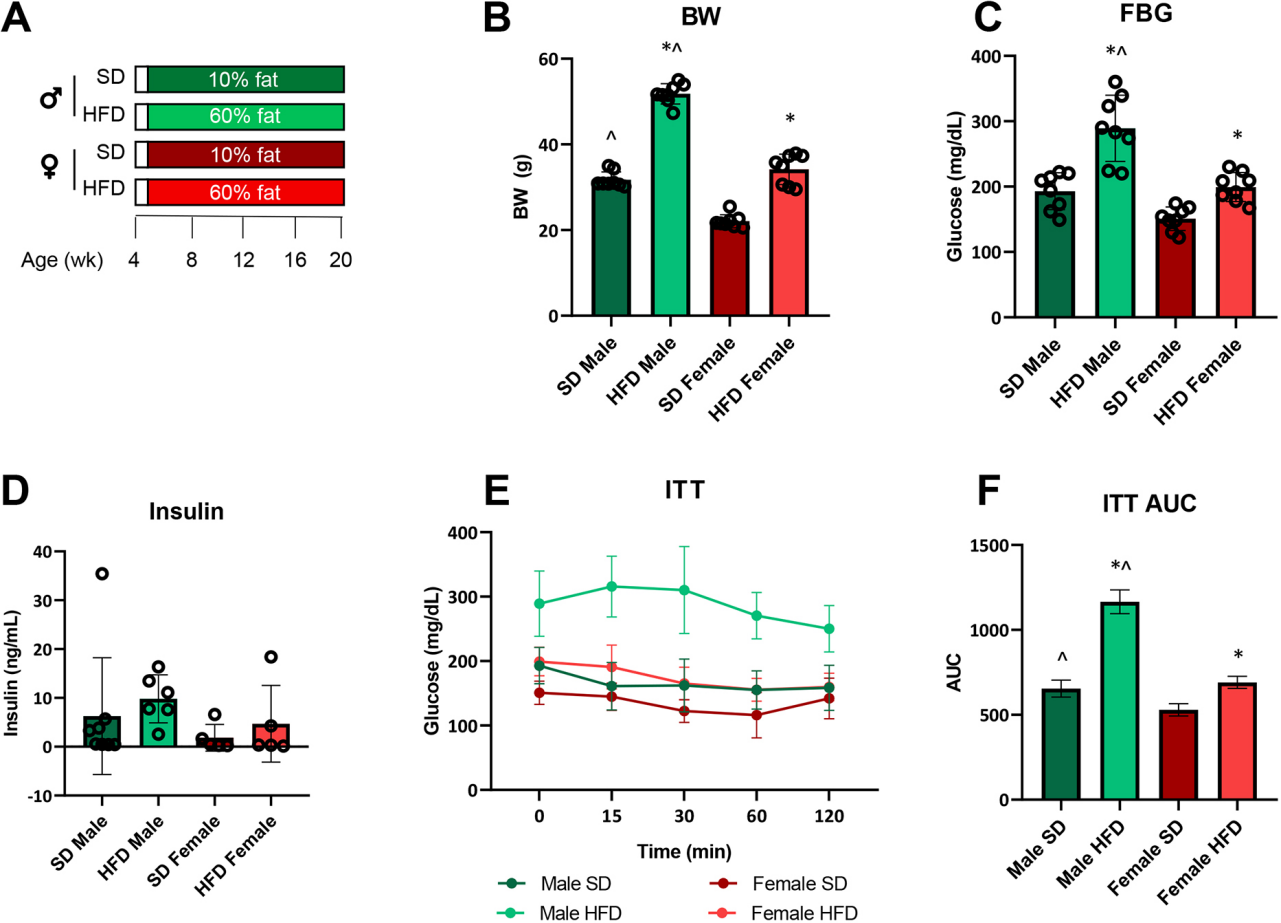
**Terminal (20 weeks) metabolic phenotyping in HFD versus SD male and female C57BL6/J mice.** (A) Study paradigm. (B) Terminal body weight (BW; *n*=8/group). (C) Terminal fasting blood glucose (FBG; *n*=8/group). (D) Terminal plasma insulin (*n*=8 SD males, *n*=6 HFD males, *n*=5 SD females, *n*=5 HFD females). (E) Terminal insulin tolerance tests (ITT; *n*=8/group). (F) Terminal ITT area under the curve (AUC; *n*=8/group). HFD, high-fat diet; SD, standard diet; individual data points represent individual animals. Statistically significant (*P*<0.05) differences from respective SD controls are represented by * and sex differences within diet by ^. Statistical analyses: data in B, C and D analyzed by one-way ANOVA; data in E analyzed by two-way repeated measures ANOVA; data in F analyzed by AUC followed by one-way ANOVA. Data are presented as mean±s.e.m.

**Fig. 5.**
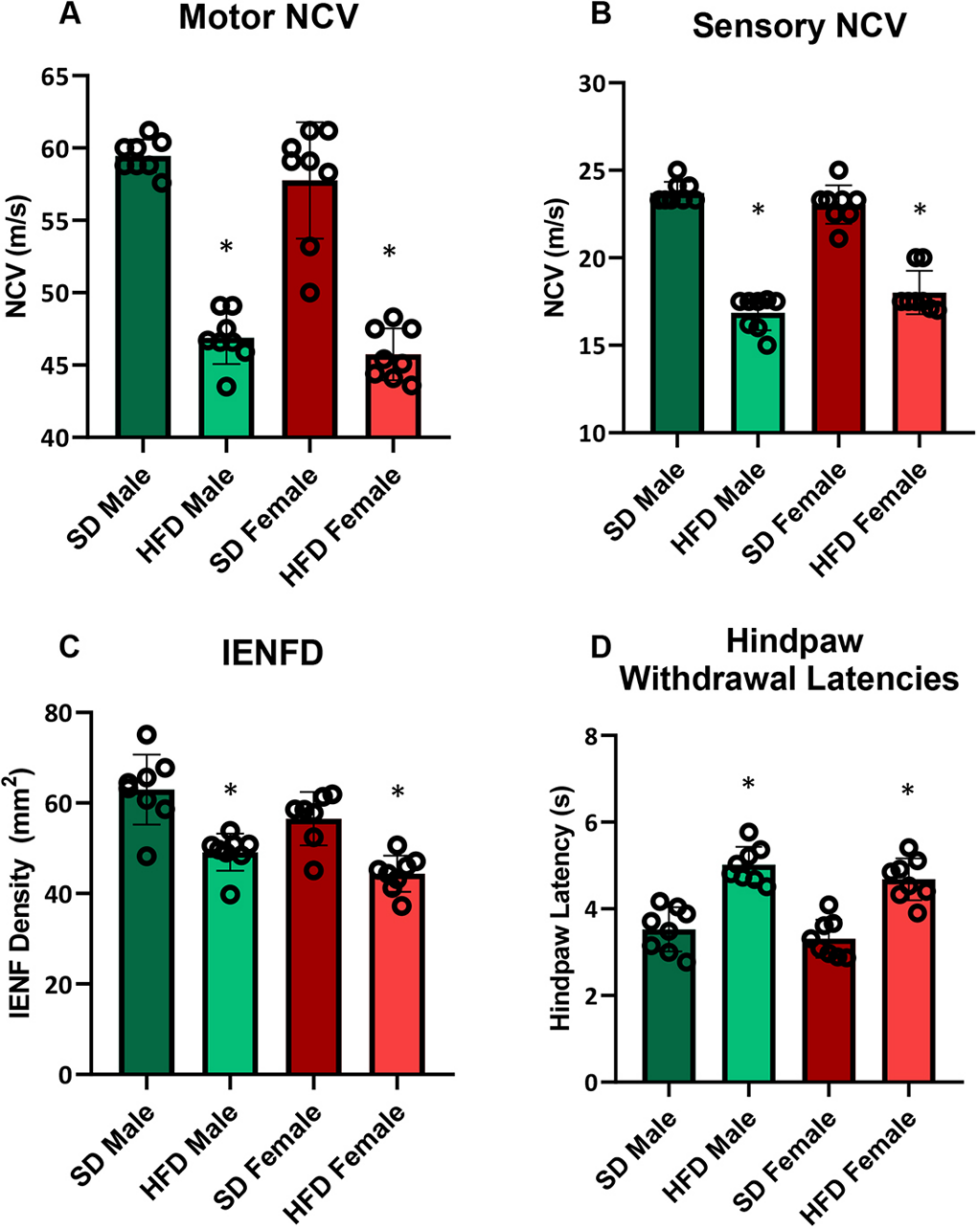
**Terminal (20 weeks) neuropathy phenotyping in HFD versus SD male and female C57BL6/J mice.** (A) Motor nerve conduction velocities (NCVs; *n*=8/group). (B) Sensory NCVs (*n*=8/group). (C) Intraepidermal nerve fiber densities (IENFDs; *n*=8 SD males, *n*=8 HFD males, *n*=7 SD females, *n*=8 HFD females). (D) Hindpaw withdrawal latencies (*n*=8/group). HFD, high-fat diet; SD, standard diet; individual data points represent individual animals. Statistically significant (*P*<0.05) differences from respective SD controls are represented by *. Statistical analyses: data in A, B and D analyzed by one-way ANOVA; data in C analyzed by non-parametric Kruskal–Wallis test. Data are presented as mean±s.e.m.

Although HFD females retain insulin sensitivity relative to HFD males (Fig. 2), they still developed NCV defects (Fig. 3A,B) and IENFD loss (Fig. 3C). Therefore, we conducted a second study, designated Cohort 2, that terminated earlier, at 20 weeks of age (i.e. after 15 weeks of diet; Fig. 4A), to examine earlier NCVs and IENFD measures at that time point. Metabolic phenotyping revealed the anticipated changes based on our initial Cohort 1 study (presented in Fig. 1). HFD females gained significantly less weight than their male counterparts (Fig. 4B), which was also the case in our initial study at the same 20 weeks milestone (Fig. 1B). Moreover, as expected, HFD females had lower FBG (Fig. 4C) and lower, but non-significant, plasma insulin (Fig. 4D) than HFD males at 20 weeks, in agreement with improved insulin sensitivity observed initially in HFD females. SD animals did not differ in FBG (Fig. 4C) or insulin (Fig. 4D) regardless of sex. Terminal ITTs at 20 weeks in Cohort 2 (Fig. 4E,F) were also in agreement with the longitudinal ITT assessments at 20 weeks from our initial study (Fig. 2A,B), which demonstrated greater insulin sensitivity in HFD females versus males. Despite sex differences in insulin sensitivity, sex did not affect motor (Fig. 5A) or sensory (Fig. 5B) NCVs nor IENFDs (Fig. 5C). Regardless of sex, SD animals had higher NCVs and IENFDs compared to HFD animals, as expected. Interestingly, sex did not affect hindpaw thermal latency at the 20 weeks time point, which was longer in HFD versus SD animals (Fig. 5D). This contrasts with the later 24 and 36 weeks time points in our initial Cohort 1 study, which found sex differences in both HFD and SD cohorts (Fig. 3D), suggesting that differences in hindpaw withdrawal latency develop at later time points in older mice.

### Sex differences in lipid and adipokine levels

Although we did not detect sex differences in early PN measures (20 weeks), HFD and SD female mice were more insulin sensitive at earlier time points, including at 20 weeks. Thus, we next assessed additional metabolic parameters related to insulin sensitivity, energy homeostasis and inflammation by quantifying circulating lipid and adipokine levels in plasma collected at the 20 weeks terminal endpoint (Table 1). There were no differences among the groups in circulating triglyceride or non-esterified fatty acid levels. However, cholesterol levels were higher in male versus their respective female dietary cohort. Sex variation was present in circulating phospholipids, which were also consistently elevated in males, independent of diet. With regards to circulating adipokine plasma levels, as anticipated, HFD (particularly in males) increased leptin levels, but leptin was not different between SD males and females. Adiponectin was highest in HFD females and lowest in HFD males, but did not differ by sex in SD-fed animals. Resistin, another energy-regulating adipokine, also differed across groups, and was lower in SD versus HFD males and in SD males versus SD females. It did not differ between HFD and SD females, nor between HFD males and females. Among the pro-inflammatory or immune-regulating cytokines, concentrations of interleukin-6 (IL-6) and monocyte chemoattractant protein 1 (MCP-1) were higher in SD females versus males, and MCP-1 was higher in HFD males versus their SD counterparts. There were no differences between male and female HFD-fed animals for either cytokine. Finally, the fibrinolysis inhibitor plasminogen activator inhibitor 1 (PAI-1; also known as Serpine1) was increased in response to HFD feeding, but only in males. SD males, SD females and HFD females all had similar PAI-1 levels.

**Table 1.**
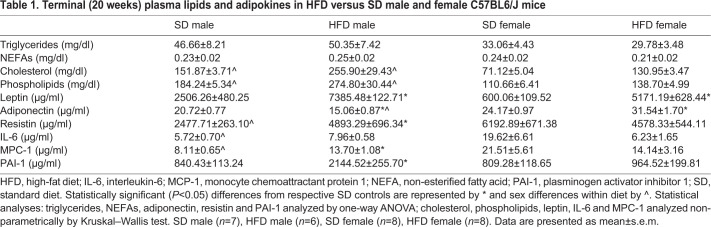
**Terminal (20 weeks) plasma lipids and adipokines in HFD versus SD male and female C57BL6/J mice**

## DISCUSSION

Animal models have significantly advanced our understanding of disease, including prediabetes, diabetes and PN (O'Brien et al., 2014). Numerous robust models exist to investigate PN pathophysiology (O'Brien et al., 2014; Hinder et al., 2017a; O'Brien et al., 2018) and test potential interventions and drug candidates (O'Brien et al., 2020; Hur et al., 2015; Eid et al., 2020; Hinder et al., 2017b). Although the precise mechanisms remain unknown, hyperglycemia, dyslipidemia and IR have been proposed to contribute to PN onset and development (Vincent et al., 2011; Savelieff et al., 2020; Grote and Wright, 2016; Stino and Smith, 2017). Unfortunately, the large majority of earlier studies solely employed male mice. In light of the potential effect of sex on disease pathophysiology, we revisited metabolic and neuropathic dysfunction by sex in the wild-type C57BL6/J HFD mouse (Table 2).

**Table 2.**
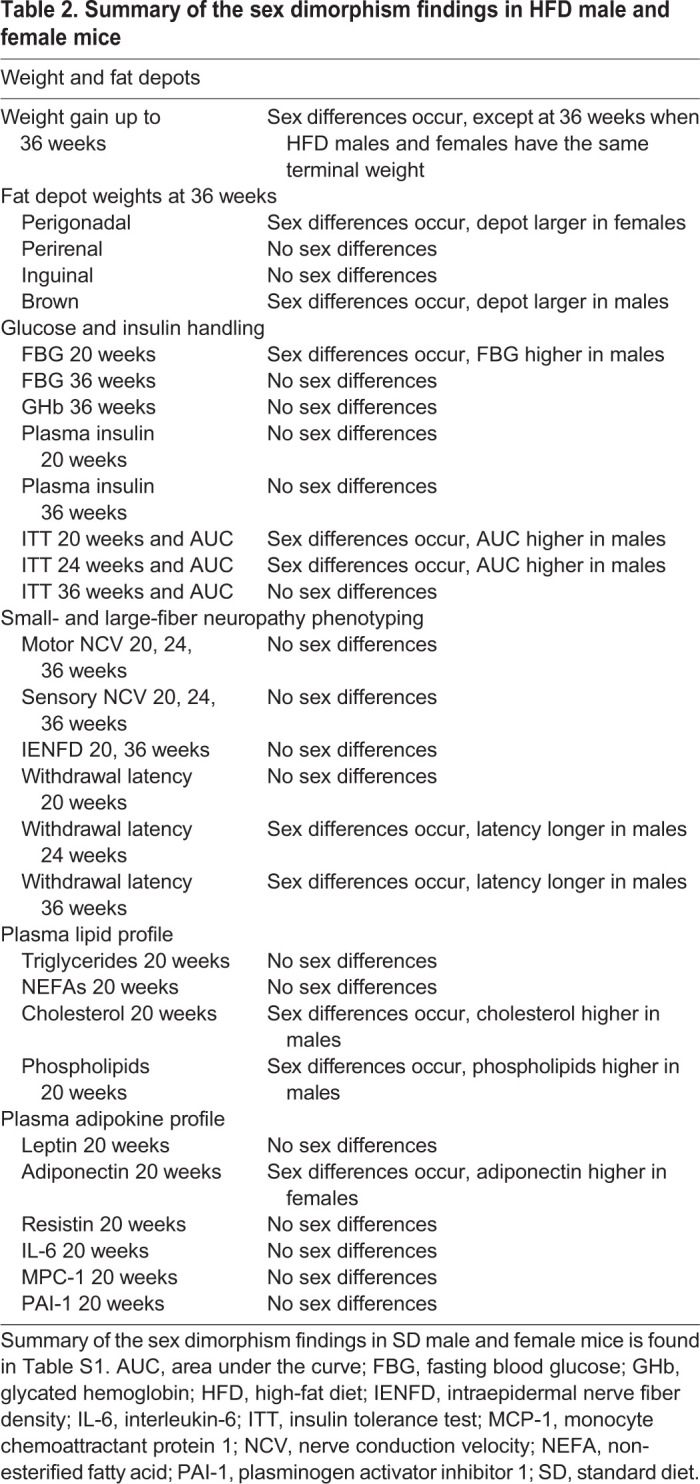
**Summary of the sex dimorphism findings in HFD male and female mice**

HFD mice were developed to model diet-induced obesity and prediabetes in humans (Singleton et al., 2005; Callaghan et al., 2020a,b), and faithfully reproduce many metabolic and neuropathic characteristics present in humans (Hinder et al., 2017a; O'Brien et al., 2018, 2020). In our current study, young mice aged 5 weeks were placed on either a SD or HFD and were monitored up to 36 weeks of age, which corresponds to the period approaching middle age in mice. During that time, both male and female HFD mice developed metabolic dysfunction, including weight gain, fat depot gains and loss of insulin insensitivity as an IR marker. However, metabolic dysfunction was delayed in females, which gained less weight and retained a more normal metabolic profile and insulin sensitivity, compared to HFD males earlier in the study time course. Despite the delay in weight gain in HFD females up to 24 weeks, their final weight was similar to HFD males at 36 weeks; therefore, HFD females gained weight more steeply than their male counterparts in the last weeks of the study as they neared middle age. This mirrors the scenario in humans, in which middle-aged women experience accelerated increase in waist circumference compared to males, presumably due to dropping estrogen levels (Kautzky-Willer et al., 2016). Moreover, although we did not measure sex hormones, a study limitation, estrogen exerts sex differential effects on the central nervous system to influence eating habits in mice (Cao et al., 2014; Saito et al., 2015; Xu et al., 2011), which could be another reason for the different initial weight gain in HFD females versus males.

As anticipated, HFD feeding decreased motor and sensory NCVs and IENFD, and increased thermal latencies, at early and later time points in both sexes compared to SD feeding (Hinder et al., 2017a; O'Brien et al., 2018, 2020). Intriguingly, despite delayed weight gain and insulin sensitivity loss, and a generally better early metabolic profile, HFD females still developed small- and large-fiber defects at the same stages as their male counterparts. The only sex differences observed were subtle effects on thermal latencies at the later (24, 36 weeks), but not earlier (20 weeks), time points. We observed no sex differences in nerve electrophysiology at 20, 24 and 36 weeks or nerve anatomy at 20 and 36 weeks. Thus, overall, the lack of effect on neuropathy was surprising considering that the pathophysiology proposed to contribute to PN, such as IR (Grote et al., 2013a,b; Grote and Wright, 2016; Kim et al., 2011) and metabolic syndrome components (Callaghan et al., 2020a, 2016b, 2018, 2016a), differed between HFD males and females in the early stages up to 24 weeks.

The sex differences in metabolic status noted among HFD animals in our study reflect clinical studies in humans. Under physiological conditions, adult human females have more protective subcutaneous adipose tissue (e.g. gluteofemoral depot) and brown adipose tissue depots compared to males, which have relatively larger inflammatory (visceral) VAT depots (Kautzky-Willer et al., 2016; Enzi et al., 1986; O'Brien et al., 2017). This sex differential distribution is shaped by estrogen levels (Power and Schulkin, 2008). We noted higher VAT (perigonadal, perirenal) in SD males versus females as expected at 36 weeks, equivalent to adult middle age in humans; however, SD males also had higher inguinal (flank) and brown adipose tissue depots. Rodents just entering middle age may experience a drop in estrogen levels (Wise, 1982), which could shift middle-aged females to a less protective fat profile, with less subcutaneous and brown adipose tissue, which also occurs in humans (Kautzky-Willer et al., 2016). This may account for aging SD female mice at 36 weeks having smaller inguinal and brown adipose tissue depots compared to SD males.

Under pathological obesogenic conditions, both human males and females gain more VAT (Kautzky-Willer et al., 2016). This effect is particularly pronounced in females transitioning from pre-menopause to post-menopause with a concomitant estrogen level drop, which consequently exhibit a stronger preferential shift to VAT compared to males, as well as a loss of insulin sensitivity (Kautzky-Willer et al., 2016). This same pattern occurs in mice and is supported here by alterations in fat deposition in our middle-aged HFD females, which gained more perigonadal and perirenal VAT and had smaller brown adipose tissue depots relative to HFD males. Although diet duration differed, others have also shown that HFD female mice accumulated more perigonadal fat compared to their male counterparts (Wu et al., 2017; Medrikova et al., 2012). Although we found that HFD females tended to have more VAT compared to their male counterparts, the 36 weeks NASH scores, which are a surrogate of ectopic liver fat deposition, were greater in HFD males versus females. This is in agreement with most human studies, although lean versus obese status somewhat tempers the relationship (Pan and Fallon, 2014; Ter Horst et al., 2015; Kautzky-Willer et al., 2016), and with a study of triglyceride accumulation in liver in HFD males and females (Medrikova et al., 2012). Therefore, our SD and HFD animals mirror the sex differences in fat deposition in humans under both physiological and pathological conditions.

A transition from a lean to an obese profile, which is characterized by VAT expansion, is linked to adverse metabolic health in both sexes, including IR (de Mutsert et al., 2018; Campos et al., 2019), dyslipidemia (Fox et al., 2007) and altered adipokine levels (Campos et al., 2019; Unamuno et al., 2018). When we examined insulin sensitivity in SD animals, males were less sensitive than females throughout the time course, and sensitivity declined for both sexes with age. In our HFD cohort, males consistently suffered from poor insulin sensitivity, already beginning at the 20 weeks time point, which paralleled weight gain starting at 16 weeks. In contrast, weight gain was more gradual in HFD females, which retained insulin sensitivity until 36 weeks, when IR differences to males were no longer present. These metabolic features mirror clinical studies, which demonstrate that lean human females, who tend to accumulate relatively more subcutaneous adipose fat versus VAT, are more insulin sensitive than lean males, but that aging and obesity erodes IR differences between sexes (Cnop et al., 2003; Kautzky-Willer et al., 2016). Thus, the SD to HFD transition in this mouse model by sex recapitulates changes in insulin sensitivity in humans by sex.

We also assessed plasma lipid and adipokine profile by sex and diet status at 20 weeks (i.e. after 15 weeks of diet). We observed higher total cholesterol and phospholipid levels, and a non-significant elevation in triglycerides, in SD male versus female mice, which closely aligns with similar human plasma lipid profiles in healthy adult males and females (Cnop et al., 2003; Wang et al., 2011). At the 20 weeks time point, HFD females had retained insulin sensitivity and gained less weight compared to HFD males, and this was reflected in their better lipid profiles, with lower total cholesterol and phospholipids, and lower, albeit not significantly, triglycerides. A study in humans found no differences in triglycerides across males and females that were healthy or had prediabetes, after adjusting for age and body mass index (Donahue et al., 2007); thus, the lack of triglyceride differences may not be surprising, and we, and others, have noted this previously in SD versus HFD comparisons (Hinder et al., 2017a; Eisinger et al., 2014; Bruder-Nascimento et al., 2017).

In our cohort, HFD had a more pronounced effect on leptin and adiponectin levels than did sex. SD mice did not differ in leptin or adiponectin levels, although healthy human females have naturally higher leptin and adiponectin levels than males (Cnop et al., 2003). Leptin and adiponectin are related to body fat content and distribution (Cnop et al., 2003; Christen et al., 2018); therefore, the SD female mice, which were very lean, may not have followed the natural pattern in humans. HFD increased leptin independent of sex, which is a well-established phenomenon in obese humans (Farr et al., 2015), and highlights a strong and important additional strength of the HFD model versus the *ob*/*ob* and *db*/*db* models, which are deficient in leptin signaling. HFD lowered adiponectin in male mice but raised it in females, whereas it increased resistin in male mice but did not affect levels in females. In humans, obesity decreases adiponectin and increases resistin in male and female cohorts (Liu et al., 2020; Christou et al., 2020), although some studies note no differences (Koster et al., 2010). In a cohort stratified by sex, adiponectin was more elevated in overweight and obese females versus males (Lew et al., 2017), as we observed in our cohort, although adjusting some clinical parameters tempered the relationship.

HFD did not universally increase adipokine inflammatory or fibrinolysis/thrombosis markers, nor was there a fixed pattern by sex status, and this is mirrored in human clinical studies of obesity and/or prediabetes (Koster et al., 2010; Donahue et al., 2007). Adipokines derive from adipose tissue and increase during obesity and fat depot expansion (Ouchi et al., 2011). Additionally, estrogen shapes adipose inflammatory and fibrotic state, and fat depot and adipocyte size (Davis et al., 2013), which could differently influence adipokine profiles in males and females. MPC-1 and PAI-1 significantly increased in HFD males, and there was a trending increase in IL-6. This was not observed in HFD females, for which there was an unexpected trending decrease in MPC-1 and IL-6, despite the fact that they had gained significantly more weight than their SD counterparts. IL-6 and MPC-1 do not differ by sex in humans in either lean, overweight or obese cohorts (Lew et al., 2017; Donahue et al., 2007), although adjusting for some clinical parameters indicates some possible difference in MPC-1 (Lew et al., 2017). PAI-1, on the other hand, does differ by sex in clinical studies. In mice, we found sex differences in IL-6 and MPC-1 in lean animals. Therefore, the HFD mouse model captures many, but not all, of the lipid and adipokine profiles in humans by obesity/prediabetes and sex. Importantly, however, there is heterogeneity in clinical findings, and adjusted multivariate models adjust potential relationships, which was not conducted in our mouse study.

Interestingly, although we noted several sex differences in metabolic parameters, there was little influence of sex on PN, despite variation in rate of weight gain, FBG, lipids and IR, which have been proposed to contribute to PN onset and development (Vincent et al., 2011; Savelieff et al., 2020; Grote and Wright, 2016; Callaghan et al., 2020a). Large-fiber electrophysiological measures of nerve function did not vary by sex in either HFD or SD cohorts. These mouse data parallel our clinical experience of sex not being a risk factor for incident PN in adult patients with diabetes (Andersen et al., 2018) or obese patients (Callaghan et al., 2020b, 2016b; Reynolds et al., 2020). We also found that sex was not a PN determinant in youth in the SEARCH cohort (Jaiswal et al., 2017). In the MONICA/KORA study in patients with diabetes, sex was not a risk factor for PN, impaired fasting glucose, impaired glucose tolerance or normal glucose tolerance (Ziegler et al., 2008). Although smaller studies found that female (Won et al., 2012) or male (Booya et al., 2005) sex was a multivariate risk factor for diabetic PN, as a whole, sex is not observed to be a PN risk factor. In cohorts in which sex is a reported variable, differences may arise from as yet unidentified population characteristics, e.g. genetics, differences in primary and secondary outcome measures, or stage of PN progression and other metabolic factors, such as prediabetes versus diabetes. However, overall, findings in human studies are paralleled by our HFD mouse model, which does not exhibit sex as a PN risk factor. This further highlights the utility of HFD mice as a preclinical model of obesity, prediabetes and PN.

In rodent studies, there are documented strain-dependent variations in PN phenotypes (O'Brien et al., 2014), and the majority of studies involved male animals. However, a handful of reports have investigated sex differences in various aspects of neuropathy (PN, autonomic) and in distinct models [type 1 diabetes (T1D), HFD]. Streptozotocin (STZ)-induced T1D rats do not exhibit any differences in tail NCV between males and females, although gonadectomy improves NCV in female but not male rats (Pesaresi et al., 2011). This observation was ascribed to differing neuroactive steroid levels in mouse sciatic nerves, which affects axonal transport through altered mitochondrial dynamics (Pesaresi et al., 2018). HFD induces cardiac autonomic nervous dysfunction in both sexes after 24 weeks of diet, although the mechanism may differ by sex (Bruder-Nascimento et al., 2017). HFD also induces gastrointestinal autonomic dysfunction independent of sex after 8 weeks of diet, even though female mice do not gain as much weight or develop IR compared to males (Nyavor et al., 2019). This observation is consistent with our current HFD study on PN, in which females develop PN within the same time frame as males, despite delayed onset of metabolic dysfunction compared to males.

IR development has been suggested to contribute to PN progression in diabetes (Grote and Wright, 2016). The sciatic nerve in wild-type C57BL/6J is responsive to insulin and activates Akt signaling (Grote et al., 2013b), which indicates that the peripheral nervous system (PNS) may become susceptible to IR. Indeed, we have shown that hyperinsulinemia induces IR in dorsal root ganglia (DRG) (Kim et al., 2011), suggestive of insulin sensitivity loss in the PNS under prediabetic conditions. In T1D, the extent of mitochondrial dysfunction *in vitro* in DRG is greater when isolated from untreated versus insulin-treated STZ-induced T1D rats (Chowdhury et al., 2010; Huang et al., 2003). In the T2D leptin-deficient *ob*/*ob* mouse, DRG and sciatic nerve exhibit impaired insulin signaling responses (Grote et al., 2013a). Therefore, our finding that delay in systemic metabolism in HFD females did not translate to a delay in PN onset was surprising. This may arise from differences in systemic metabolism versus the local nerve microenvironment, which may be more susceptible to IR development, as seen in our recent study (O'Brien et al., 2020). Because dyslipidemia is a global IR feature, we recently performed lipidomic and transcriptomic analyses of sciatic nerves from HFD mice with PN (O'Brien et al., 2020). We observed an increase in triglycerides, which contained saturated fatty acids and correlated with transcriptomic evidence of lipid pathway dysregulation. We discovered a specific increase in diacylglycerol acyltransferase 2, the enzyme required for the final committed step of triglyceride synthesis. We validated this finding in human sural nerve biopsies from diabetic patients with PN, confirming that abnormal nerve-lipid signaling, a critical factor in IR, contributes to peripheral nerve dysfunction in both prediabetes and T2D (O'Brien et al., 2020). Thus, nerves in both male and female mice may develop IR to a similar extent earlier during HFD feeding, even at stages when HFD females have retained greater systemic insulin sensitivity compared to males.

Despite the lack of sex differences in electrophysiological nerve measures in this mouse study, we did observe that sex influenced behavioral responses to a heat stimulus at later, but not earlier, time points. Generally, PN progression presents with sensory symptoms that range from prickling and burning to numbness, and nerve fiber damage, which ultimately leads to loss of sensation (Feldman et al., 2019). Here, HFD mice of both sexes met the defined electrophysiological and anatomical features of PN (Jolivalt et al., 2016), with slowed NCV, decreased IENFD defects and prolonged thermal latencies. However, females regardless of diet had shorter thermal latencies compared to their male counterparts at 24 and 36 weeks of age, although there were no differences in sensory NCV. Our findings align with a study of HFD obese mice, in which sex impacted facial pain response to heat (Rossi et al., 2013), but differs from a rat study of a HFD-STZ model of T2D, which reported no sex differences in either mechanical, heat or cold responses (Ferhatovic et al., 2013). With respect to human studies, our finding of shorter thermal latencies in female animals, a sign of hyperalgesia, correlate with clinical studies, in which neuropathic pain is more frequent (Abraham et al., 2018; Abbott et al., 2011; Cardinez et al., 2018) or intense (Abraham et al., 2018) in females compared to males in T2D populations.

Our study has some limitations. We may not have been sufficiently powered to detect sex differences within dietary groups, although the study was powered to detect HFD versus SD effects in males, based on our previous findings (Hinder et al., 2017a; O'Brien et al., 2018, 2020). We repeatedly assessed metabolic and neuropathy parameters longitudinally (e.g. body weight, insulin sensitivity, motor and sensory NCVs, and withdrawal latency to heat) and IENFDs at two terminal time points (20 weeks and 36 weeks); however, we only performed one terminal time point for fat depot mass (36 weeks) and circulating lipid and adipokine levels (20 weeks). Furthermore, we did not measure estrogen, testosterone or other endocrine marker levels. Although we noted no sex PN differences, sex hormones exert an impact on feeding behavior centrally and shape adipose tissue (Wang and Xu, 2019; Davis et al., 2013); therefore, it was an important study limitation. Lastly, this study focused on prediabetes and did not consider effects in diet-induced T2D models. All these factors should be considered in future studies of sex differences in PN.

In summary, in our HFD-induced model of obesity, prediabetes and PN, we found that although HFD females exhibit a delay in IR development and weight gain compared to HFD male mice, they develop comparable PN within the same time frame. An exciting future avenue will be to understand why an improved metabolic profile in obese and prediabetic female mice does not protect from PN (Vincent et al., 2011; Savelieff et al., 2020; Grote and Wright, 2016; Stino and Smith, 2017). This may arise from several factors, including differing fat accumulation and distribution or circulating lipid or adipokine concentrations. Another possibility is that nerves may be especially susceptible to HFD and nutrient overload compared to other tissues; therefore, although we noted a delay in systemic metabolic dysfunction in females, the nerve metabolic milieu may already be impaired to similar extents in males and females early upon HFD feeding, leading to PN within the same timeframe in both sexes (O'Brien et al., 2020). These new areas of investigation will lead to new insights in PN pathogenesis. Collectively, our data suggest a complex relationship between metabolism and metabolic factors and sex in PN. Our results also suggest that the HFD mouse continues to be a good model of diet-induced obesity and prediabetes, especially when sex is considered as a variable.

## MATERIALS AND METHODS

### Experimental animals

Male and female C57BL/6J mice aged 4 weeks (wk) were purchased from The Jackson Laboratory (Bar Harbor, ME, USA; catalog #000664). Animals were housed in a specific pathogen-free suite kept at a temperature of 20±2°C with a 12 h:12 h light-dark cycle at the Unit for Laboratory Animal Medicine (ULAM) at the University of Michigan. Mice were limited to a maximum of five littermates per cage to avoid fighting and aggressive behavior. They had *ad libitum* access to drinking water and food, were housed on fine corncob bedding and provided nestlets for enrichment. Estrous cycles in female mice were not synchronized. Animals were monitored daily by veterinary staff. After acclimatizing for 1 week, cages were randomly allocated to SD or HFD groups. The SD contained 10% energy from fat, 70% energy from carbohydrates and 20% energy from protein (catalog #D12450B, Research Diets, New Brunswick, NJ, USA). The HFD contained 60% energy from fat, 20% energy from carbohydrates and 20% energy from protein (catalog #D12492, Research Diets). All experimental protocols were approved by the University of Michigan's Institutional Animal Care and Use Committee (IACUC) (PRO00006140 from 02/10/2015 to 02/10/2018).

Experiments were performed on two separate cohorts. Cohort 1, ‘36 wk study paradigm’, mice were fed SD or HFD starting at 5 weeks of age until 36 weeks of age. Cohort 2, ‘20 wk study paradigm’, were fed SD or HFD starting at 5 weeks of age until 20 weeks of age. Cohort 1 underwent longitudinal and terminal metabolic and neuropathic phenotyping. Cohort 2 underwent terminal metabolic and neuropathy phenotyping and were assessed for terminal circulating lipid and adipokine levels. Mice were transported to designated procedure rooms within the animal facility for all metabolic and neuropathic assessments, which were performed between 09:00 and 17:00. At study conclusion, mice were euthanized by pentobarbital overdose [150 mg/kg intraperitoneal injection (i.p.); Fatal-Plus, Vortech Pharmaceuticals, Dearborn, MI, USA]. All study procedures complied with Diabetic Complications Consortium (www.diacomp.org) protocols and were approved by the University of Michigan IACUC.

### Metabolic phenotyping

FBG levels were measured starting at 8 weeks of age and then every 4 weeks to document the onset and progression of hyperglycemia. FBG was recorded after a 4 h fast by analyzing one drop of tail blood using a glucometer (AlphaTRAK, Abbott Laboratories) with compatible glucose test strips (Zoetis, Parsippany, NJ, USA). For ITTs, 0.075 U/kg insulin (Novolin 70/30, Novo Nordisk) was injected intraperitoneally and blood glucose levels were recorded serially at 15, 30, 60 and 120 min. Insulin-injected mice that suffered hypoglycemic shock were rescued with 20% D-glucose solution (i.p.). At study end, mice were fasted, euthanized and their plasma (Cohorts 1 and 2), fat depots (perigonadal, perirenal, inguinal, neck brown adipose fat; Cohort 1) (Bagchi and MacDougald, 2019) and liver (Cohort 1) were collected. Plasma insulin levels were assessed at the Mouse Metabolic Phenotyping Center (MMPC; www.mmpc.org) at Vanderbilt (Vanderbilt University School of Medicine, Nashville, TN, USA).

Plasma cholesterol and triglyceride lipoprotein profiles were assessed by fast protein liquid chromatography (FPLC) analysis at the Cincinnati MMPC (University of Cincinnati Medical Center, Cincinnati, OH, USA). Plasma adipokine levels were assessed by custom array for adiponectin, leptin, IL-6, MCP-1, PAI-1, resistin and tumor necrosis factor alpha (TNF-α; also known as TNF) (Eve Technologies, Calgary, Canada). Fat depots were weighed. Livers were evaluated for NASH by the University of Michigan ULAM *In Vivo* Animal Core. Briefly, histopathology on liver from mice at 36 weeks of age was performed without knowledge of the experimental groups by a board-certified veterinary pathologist. Liver sections were stained with Hematoxylin and Eosin and scored using non-alcoholic fatty liver disease activity score and steatosis score, as previously described (Kleiner et al., 2005).

### Neuropathy phenotyping

Neuropathy phenotyping was performed as previously described (O'Brien et al., 2020, 2018), according to guidelines issued by the Diabetic Complications Consortium (www.diacomp.org).

#### Hindpaw withdrawal latency

Hindpaw withdrawal latency to a heat stimulus was measured using an analgesia meter (336TG model, IITC Life Sciences, Woodland Hills, CA, USA). Mice were placed in acrylic compartments on a warm (32°C) glass plate and allowed to habituate for 45 min. The heat source was maneuvered under the hindpaw and its temperature was set to 25°C. The temperature was increased to 55°C over the course of 20 s, and the time from beam activation to the time of paw withdrawal was recorded. A maximum threshold time limit of 20 s was applied to prevent injury to the mice. Hindpaw stimuli were alternated between each foot, with ∼10 min between each individual stimulus. Six measurements were taken per mouse and an average latency time was calculated.

#### Nerve conduction studies

Isoflurane (Hospira, Lake Forest, IL, USA) was used to anesthetize mice, which were induced with a 4-5% dose and maintained at 1-2%. The animals’ core temperature was held at 34°C with a heating lamp. Anesthesia onset was judged by impaired righting reflex and decreased pedal withdrawal. Using stainless steel needle electrodes (Natus Medical, Pleasanton, CA, USA), sural sensory NCVs were recorded at the foot dorsum following antidromic supramaximal stimulation at the ankle. Sural sensory NCVs were calculated by dividing the distance by the sensory nerve action potential take-off latency. Sciatic-tibial motor NCVs were recorded at the foot dorsum following orthodromic supramaximal stimulation, first at the ankle then at the sciatic notch. Latencies were measured in each case from the initial onset of the compound muscle action potential. Sciatic-tibial motor NCVs were calculated by subtracting the measured ankle distance from the measured notch distance. The resultant distance was then divided by the difference in ankle and notch latencies for a final motor NCV.

#### IENFD

Prior to perfusion, foot pads were collected from the hindpaw plantar surface, immersed in Zamboni Fixative (catalog #1459A, Newcomer Supply, Middleton, WI, USA) overnight at 4°C, rinsed in 5, 10, and 20% sucrose in 0.1 M sodium phosphate buffer (pH 7.4), cryoembedded in OCT compound (Tissue-Tek, Sakura Finetek, Torrance, CA, USA) and sectioned (30 μm). Sections were incubated with primary antibody against the pan-axonal marker, PGP9.5 (also known as UCHL1; 1:2000 primary antibody; catalog #14730-1-AP, Proteintech, Rosemont, IL, USA), rinsed and stained with secondary antibody (goat anti-rabbit, highly cross-adsorbed Alexa Fluor 488; catalog #A-11008, Invitrogen, Thermo Fisher Scientific, Waltham, MA, USA). Three images per mouse (over a 3 mm depth) were collected on an Olympus FluoView 500 confocal microscope using a 20×/1.2 NA objective at a resolution of 1024×1024 pixels. The optical section thickness was 3.3 μm. Ten images per stack were flattened using the maximum project arithmetic option in MetaMorph (version 7.7.0.00, Molecular Devices, San Jose, CA, USA). Fiber counts and lengths were quantified by a blinded investigator and the data are presented as the number of fibers per millimeter.

### Statistical analysis

Sample sizes of *n*=8 per group are adequately powered to detect significant differences based on previously published studies (Vincent et al., 2009; Hinder et al., 2017a). Analyses were performed using Prism 8 (GraphPad, La Jolla, CA, USA). Normality of residuals was determined using Anderson–Darling, D'Agostino-Pearson omnibus, Shapiro–Wilk and Kolmogorov–Smirnov tests. For normally distributed data, statistically significant differences (*P*<0.05) were determined using ANOVA (one or two-way, with the used of repeated measures for temporal data) or mixed model, with Tukey's post-test for multiple comparisons. For non-normally distributed data, datasets were analyzed non-parametrically using the Kruskal–Wallis test with Dunn's post-test for multiple comparisons. Data are presented as mean±s.e.m., unless otherwise stated.
